# Short-term clinical efficacy of five-step manipulation under anaesthesia combined with arthroscopic shoulder surgery for adhesive capsulitis

**DOI:** 10.3389/fsurg.2025.1724309

**Published:** 2026-01-09

**Authors:** Weiqing Zeng, Ziqi Jin, Hongmei Li, Liangji Zhou, Yongxing Tan, Sheng Chai, Qing Lü, Hua Wei, Gangjian Tang

**Affiliations:** 1 Department of Orthopaedics, Guilin Municipal Hospital of Traditional Chinese Medicine, Guilin, Guangxi, China; 2 Graduate College, Guangxi University of Chinese Medicine, Nanning, Guangxi, China; 3 Department of Blood Transfusion, Suining Central Hospital, Suining, Sichuan, China

**Keywords:** adhesive capsulitis, arthroscopic capsular release, manipulation under anaesthesia, observational study, shoulder arthroscopy

## Abstract

**Objective:**

To compare the short-term efficacy of arthroscopic capsular release combined with five-step manipulation under anaesthesia vs. five-step manipulation under anaesthesia alone for adhesive capsulitis.

**Methods:**

This retrospective study included 66 patients assigned to arthroscopic capsular release (ACR) plus five-step manipulation under anaesthesia (MUA) (*n* = 31) or five-step MUA alone (*n* = 35); both cohorts received intra-articular “cocktail” irrigation. Outcome assessments comprised American Shoulder and Elbow Surgeons (ASES) scores and Visual Analogue Scale (VAS) pain scores at 1 week, 1 month, and 6 months post-operatively; peri-operative metrics included operative time, length of post-operative hospital stay, and total hospitalisation cost.

**Results:**

One week and one month post-operatively, VAS scores in the two groups were similar (*P* > 0.05). At 6 months, the treatment group exhibited significantly lower VAS scores than the control group (1.83 ± 0.70 vs. 2.55 ± 0.56, 95% CI: −1.03 to −0.41, *P* < 0.001). ASES scores did not differ between groups at 1 week or 1 month (*P* > 0.05), but were significantly higher in the treatment group at 6 months (76.68 ± 6.67 vs. 73.43 ± 2.54, 95% CI: 1.42–5.32, *P* = 0.009). No complications occurred in either group. Operative time, postoperative hospitalisation days and hospitalisation cost were higher in the treatment group (*P* < 0.05).

**Conclusion:**

Compared with simple five-step MUA, the combination therapy of five-step MUA and ACR provides superior pain relief and higher ASES functional scores at 6 months after surgery. However, operation time, postoperative hospitalisation days and hospitalisation costs correspondingly increase. Due to the small sample size and short clinical observation time, further follow-up is needed for long-term efficacy.

## 1 Introduction

Frozen shoulder (FS), also termed adhesive capsulitis (AC), was first described by Codman in 1934 (1). Epidemiological studies estimate the global annual incidence of frozen shoulder at 0.35%–5%, with the US prevalence alone exceeding 0.35%, and consistently show higher rates in individuals aged 40–60 years and a notable female predilection (2, 3). The condition is characterised by a prolonged clinical course, significant pain, and severe impairment of patients’ quality of life, imposing substantial burdens on both families and society (4, 5). The aetiology and pathological mechanisms of FS remain unclear (6). The underlying pathological changes involve periarticular inflammatory reactions (7), followed by secondary capsular fibrosis (8), which reduces the glenohumeral joint cavity volume (9). Notably, the subacromial space is disproportionately affected, with adhesions between the rotator cuff interval, coracohumeral ligament, and joint capsule exacerbating motion restriction (10). These pathological changes culminate in progressive loss of passive range of motion, particularly in external rotation, abduction, and flexion (11).

Despite advances in non-surgical management (e.g., physical therapy, corticosteroid injections, and hydrodilatation), 20%–30% of patients develop refractory FS requiring surgical intervention (12, 13). Among these, manipulation under anaesthesia (MUA) combined with arthroscopic capsular release (ACR) has emerged as a promising strategy. MUA mechanically disrupts intra-articular adhesions via controlled joint distraction, while ACR targets residual fibrotic tissue through precise capsular incisions (14, 15). However, conventional MUA carries risks of humeral fractures, rotator cuff tears, or inadequate release (16), whereas standalone ACR may not fully address extra-articular adhesions.

To optimise outcomes, our study innovatively integrates five-step MUA under anaesthesia with arthroscopic shoulder surgery, aiming to synergistically release both intra- and extra-articular adhesions while minimising iatrogenic injury. Preliminary data suggest this combined approach improves functional recovery and pain relief, but rigorous clinical validation is lacking. This study evaluates the short-term efficacy of this integrated technique, comparing outcomes between treatment and control groups using validated metrics (e.g., ASES score, VAS pain score, and postoperative recurrence situation measurements).

## 2 Materials and methods

### 2.1 General information

This retrospective study was approved by the Ethics Committee of Guilin Municipal Hospital of Traditional Chinese Medicine (Approval No. 2022-XJS-001-78), with a waiver of informed consent due to anonymized data and minimal risk. All study participants were inpatients from the Department of Orthopaedics at Guilin Municipal Hospital of Traditional Chinese Medicine, affiliated with Guangxi University of Chinese Medicine, recruited between January 2020 and December 2022.

### 2.2 Inclusion and exclusion criteria

Inclusion Criteria: 1. Met the diagnostic criteria for frozen shoulder (adhesive capsulitis) as defined by the 2011 *Shoulder Stiffness* guidelines. Patients were included if they presented with insidious onset of shoulder pain and progressive stiffness in the absence of a specific inciting traumatic event. Physical examination revealed restricted active and passive range of motion in multiple planes, particularly external rotation and abduction, with passive limitation typically exceeding active limitation, consistent with capsular origin. Standard anteroposterior radiographs of the shoulder were obtained to exclude alternative pathologies such as osteoarthritis, rotator cuff tear, fracture, or dislocation. Additional imaging modalities such as magnetic resonance imaging (MRI) and x-rays were used to assess for features suggestive of capsular pathology and to rule out other causes of secondary stiffness. 2. Completion of at least three months of standard conservative management—comprising oral non-steroidal anti-inflammatory drugs (NSAIDs), physiotherapy, and supervised functional rehabilitation exercises—and good compliance with the prescribed treatment protocol. 3. Aged 30–75 years. 4. Provided written informed consent.

Exclusion Criteria: 1. Poor compliance or premature withdrawal from treatment. 2. Patients with psychiatric disorders. 3. Severe osteoporosis (T-score ≤−2.5). 4. Contraindications to anaesthesia, including cardiovascular, cerebrovascular, haematologic, hepatic, or renal diseases, hypoproteinaemia, and immunocompromised status.

### 2.3 Surgical techniques

#### 2.3.1 ACR combined with five-step MUA (treatment group)

**Figure 1:**
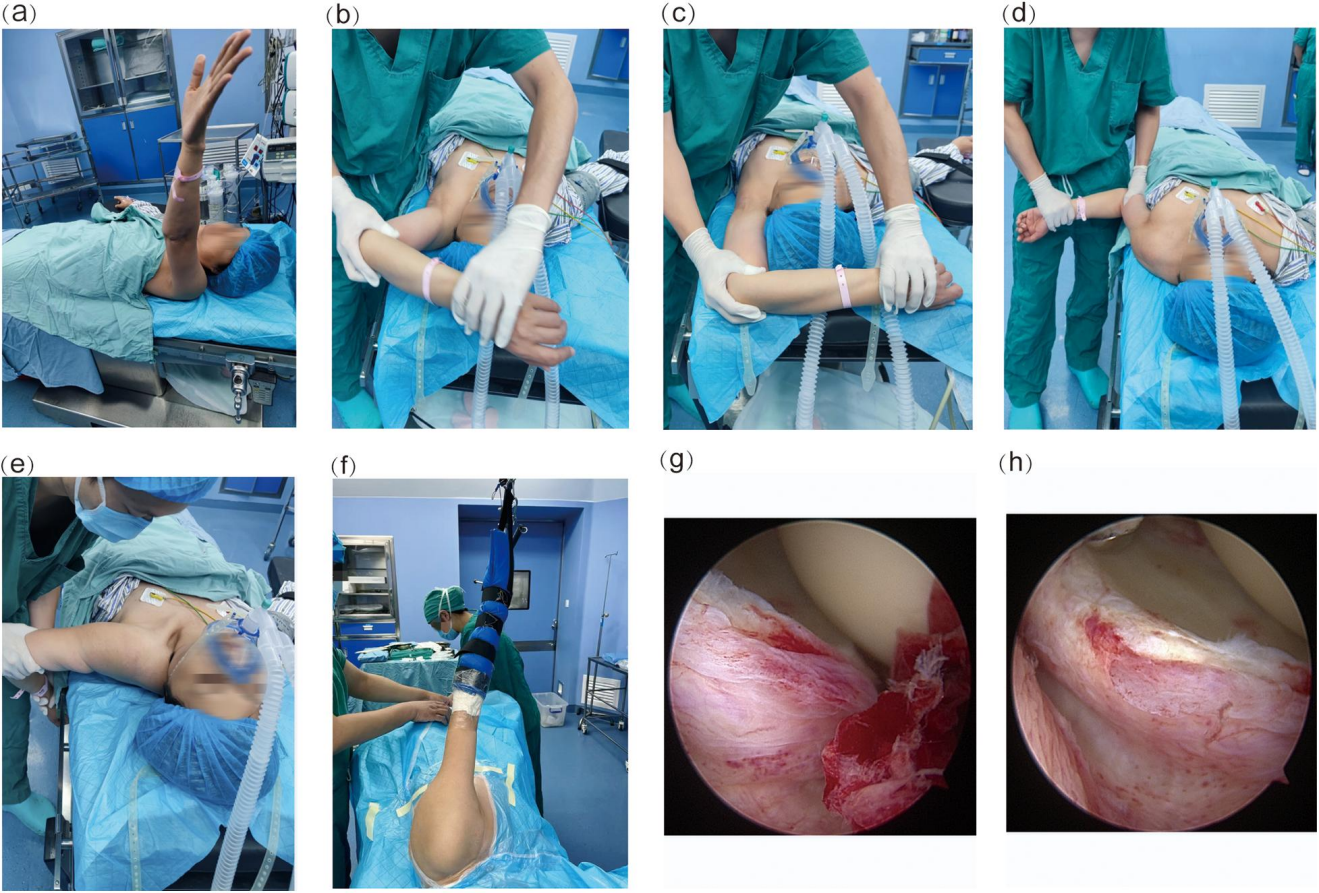
Typical case: patient, female, 54 years old, left shoulder periarthritis, treated with five-step manipulation under anaesthesia combined with arthroscopic capsular release. **(a)** The forearm of the affected limb is raised. **(b)** Passive abduction and external rotation of the affected shoulder. **(c)** Passive abduction, overhead, and external rotation of the affected shoulder. **(d)** Adduction and external rotation of the affected shoulder. **(e)** The affected shoulder extends backward to touch the back. **(f)** Side lying upper limb suspension of shoulder arthroscopic position. **(g)** Before arthroscopic release of the shoulder joint. **(h)** After arthroscopic release of the shoulder joint.

The patient is positioned supine. After marking and disinfecting the painful points around the shoulder girdle and upon successful intravenous anaesthesia, taking the release of the left shoulder as an example, the operator stands on the patient's left side. The operator firmly grasps the patient's left elbow with the left hand while palpating and protecting the left shoulder with the left hand. The shoulder is then gently and rhythmically mobilised 3–5 times through one continuous, five-step MUA cycle: elevation → abduction with external rotation → overhead abduction with external rotation → adduction with external rotation → posterior extension with the hand moving toward the back. Crepitus, or audible “clicking” sounds, may be perceived in the shoulder joint during the release procedure. Furthermore, the improvement in the shoulder joint's range of motion (ROM) is assessed. After the five-step manual release, the patient is placed in the lateral decubitus position. The shoulder joint is examined arthroscopically, with haemostasis performed using a radiofrequency knife within the joint cavity. Synovium and proliferative tissues are debrided. The anterosuperior joint capsule, posteroinferior joint capsule, posteroinferior capsular adhesions, and inferior glenohumeral ligament are sequentially released. Adhesions between the peripheral joint capsule and rotator cuff are lysed. Necessary subacromial release and acromioplasty decompression are performed. Focused release is conducted in the direction of the joint with suboptimal range-of-motion improvement after manual release (Figure 1). Finally, intra-articular and periarticular “cocktail” perfusion is administered. The cocktail consists of betamethasone sodium phosphate for Injection 4 mg (H20080565), Ropivacaine Hydrochloride Injection 150 mg/20 ml (H20060137), gentamicin sulphate injection 2 ml (H41020318) and Normal Saline 20 ml.

#### 2.3.2 Simple five-step MUA (control group)

Simple five-step manipulation under anaesthesia and cocktail therapy in the same treatment group.

### 2.4 Postoperative management and evaluation of curative effect

After the patient regains consciousness from anaesthesia, both groups are instructed to perform daily active shoulder elevation, posterior extension, and internal rotation exercises. The patient should orally take Compound Diclofenac Sodium Capsules 75 mg once daily (J20050064). Discharge is planned on postoperative days 2–6. Oral medication is continued for 2 weeks post-operation, and the patient should continue rehabilitation exercises at home after discharge.

Functional recovery following surgery was assessed as the primary outcome, using the American Shoulder and Elbow Surgeons (ASES) score at 1 week, 1 month, and 6 months postoperatively. Secondary outcomes included postoperative pain intensity measured by the Visual Analogue Scale (VAS) at the same time points, operative time, length of hospital stay, and the incidence of complications as well as recurrence of scapulohumeral periarthritis during follow-up. Patients were prospectively followed to document these endpoints and to ensure comprehensive assessment of both efficacy and safety. Recurrence was defined as the reappearance of clinically significant pain (VAS ≥ 4) and functional impairment (ASES ≤70) within 6 months postoperatively, confirmed by physical examination within 6 months postoperatively, aligning with established thresholds for moderate pain and disability in shoulder disorders (17).

### 2.5 Statistical analysis

Data were analysed using SPSS version 22.0. Normality of continuous variables was assessed by the Shapiro–Wilk test (*α* = 0.05). Variables conforming to a normal distribution were expressed as mean ± standard deviation (SD) and compared between groups using independent samples t-tests; non-normally distributed variables were described as median (interquartile range, IQR) and compared using the Mann–Whitney U test. Categorical variables were presented as frequencies (percentages) and compared between groups by the chi-square (*χ*^2^) test. Repeated-measures data (changes in VAS and ASES scores over time) were analysed by repeated-measures analysis of variance (RM-ANOVA); when the assumption of sphericity was violated (Mauchly's test, *P* < 0.05), Greenhouse–Geisser correction was applied. *post hoc* pairwise comparisons were adjusted for multiple testing using the Bonferroni method, with the adjusted significance threshold set at *α*′ = 0.05 divided by the number of comparisons. All tests were two-sided, and statistical significance was defined as *P* < 0.05.

## 3 Results

A total of 66 patients with adhesive capsulitis were screened and allocated to two surgical strategies: five-step MUA combined with ACR (treatment group, *n* = 31) or five-step MUA alone (control group, *n* = 35). Baseline characteristics are summarised in Table 1. Sex distribution (male/female: 10/21 vs. 13/22, *P* = 0.875), age (47.45 ± 7.25 years vs. 47.63 ± 7.30 years, *P* = 0.922), BMI (30.97 ± 3.00 kg/m^2^ vs. 31.64 ± 2.57 kg/m^2^, *P* = 0.330), affected side (left/right: 10/21 vs. 15/20, *P* = 0.528) and symptom duration (24.22 ± 8.14 months vs. 23.12 ± 9.40 months, *P* = 0.617) were comparable between the two cohorts, indicating balanced baseline comparability (Table 1).

**Table 1:** Basic data of two groups of patients.

Variables	Treatment group	Control group	T-value	*P*-value
Sex (male/female)	10/21	13/22	–	0.875
Age (years)	47.45 ± 7.25	47.63 ± 7.30	−0.099	0.922
BMI (years)	30.97 ± 3.00	31.64 ± 2.57	−0.982	0.330
Shoulder joint (left/right)	10/21	15/20	–	0.528
Medical history(months)	24.22 ± 8.14	23.12 ± 9.40	0.503	0.617

*Significant difference between the two groups (*P* < 0.05).

**Figure 2:**
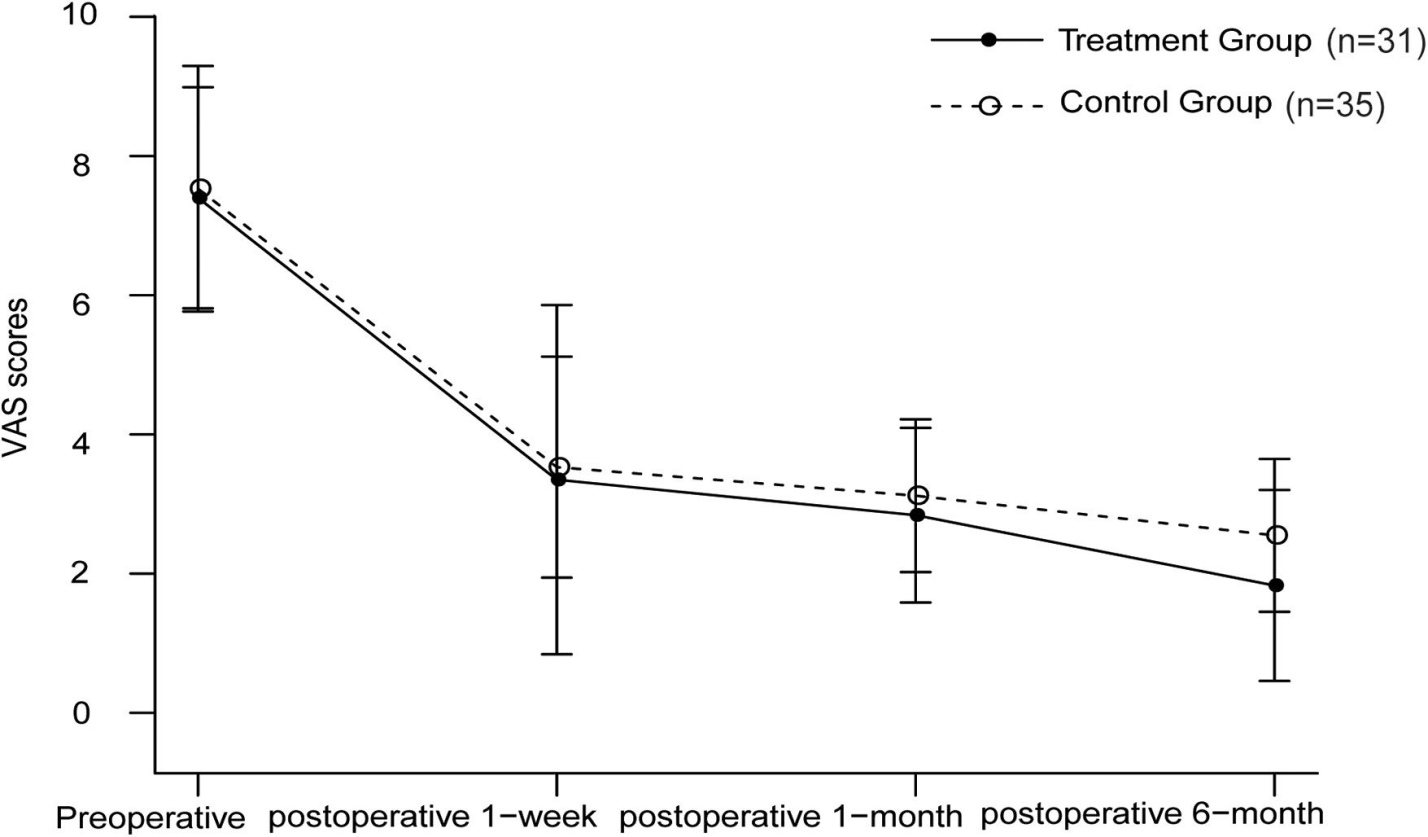
Changes in visual analogue scale (VAS) scores before and after treatment in the two groups.

One week and one month postoperatively, the reduction in VAS scores in the treatment group was comparable to that in the control group, with no statistically significant difference between the two groups (*P* > 0.05). At six months postoperatively, the VAS scores of the treatment group were significantly lower than those of the control group, and the intergroup comparison showed a statistically significant difference (1.83 ± 0.70 vs. 2.55 ± 0.56, 95% CI: −1.03 to −0.41, *P* < 0.001) (Figure 2). The ASES scores of the two groups were similar at one week and one month postoperatively (*P* > 0.05). However, at six months postoperatively, the ASES scores of the treatment group were significantly higher than those of the control group, and the difference between the two groups was statistically significant (76.68 ± 6.67 vs. 73.43 ± 2.54, 95% CI: 1.42–5.32, *P* = 0.009). The detailed data are presented in Table 2.

**Table 2:** Comparison of each scoring index between preoperative and last postoperative follow-up ($\bar{x}\pm s$).

Metric	*n*	Preoperative	Postoperative 1-week	Postoperative 1-month	Postoperative 6-month
ASES					
Treatment Group	31	37.59 ± 2.23	58.81 ± 2.34	70.21 ± 1.90	76.68 ± 6.67
Control Group	35	37.41 ± 2.00	59.15 ± 2.39	70.16 ± 1.61	73.43 ± 2.54
T-value		0.34	−0.59	0.12	2.68
*P*-value (95%CI)		0.73	0.55	0.91	*P* = 0.009^a^ (95%CI:1.42–5.32)
VAS					
Treatment group	31	7.40 ± 0.81	3.35 ± 1.28	2.84 ± 0.64	1.83 ± 0.70
Control group	35	7.53 ± 0.90	3.53 ± 0.81	3.12 ± 0.56	2.55 ± 0.56
T-value		−0.597	−0.66	−1.84	−4.61
*P*-value (95%CI)		0.55	0.51	0.07	*P* < 0.001^a^ (95%CI:−1.03 to −0.41)

a Significant difference between two groups.

Intraoperative complications (fractures, infections, nerve injury) were absent in all patients. All patients completed the 6-month follow-up. Two control-group shoulders (5.7%) experienced recurrence: one patient was readmitted at 2 months because of persistent pain attributed to incomplete release and insufficient rehabilitation, and another relapsed at 6 months. One shoulder (3.2%) in the treatment group recurred. The difference in recurrence rate was not statistically significant (*P* = 1.000). Operative time (52.74 ± 7.65 min vs. 27.77 ± 4.17 min), postoperative length of stay (5.45 ± 1.79 days vs. 2.11 ± 0.68 days) and total hospitalisation cost (CNY 14 182.23 ± 1208.00 vs. CNY 7,500.26 ± 768.06) were all significantly higher in the treatment group (all *P* < 0.001) (Table 3).

**Table 3:** Comparison of hospitalization-related indicators and recurrence rates ($\bar{x}\pm s$).

Group	*n*	Operation time (min)	Postoperative hospital stay (days)	Inpatient costs (rmb)	Postoperative Recurrence Situation
Treatment group	31	52.74 ± 7.65	5.45 ± 1.79	14,182.23 ± 1,208.00	1/31
Control group	35	27.77 ± 4.17	2.11 ± 0.68	7,500.26 ± 768.06	2/35
T-value		16.73	10.27	27.13	–
*P*-value		*P* < 0.001^a^	*P* < 0.001^a^	*P* < 0.001^a^	–

a Significant difference between two groups.

## 4 Discussion

This retrospective study analysed and compared the short-term clinical outcomes of arthroscopic capsular release combined with five-step MUA vs. five-step MUA alone for the treatment of adhesive capsulitis (frozen shoulder). Although both therapeutic approaches effectively managed adhesive capsulitis and restored shoulder function, the combination therapy (arthroscopic release plus MUA-GA) demonstrated a significantly lower recurrence rate and superior shoulder functional scores at the 6-month postoperative follow-up compared to MUA-GA alone. However, this enhanced efficacy was achieved at the cost of increased operative duration, prolonged postoperative hospital stay, and higher total hospitalisation costs.

With the rapid advancements in medicine, an increasing array of treatment modalities for scapulohumeral periarthritis has emerged. Cao *et al*. (18) conducted a systematic review and meta-analysis on extracorporeal shock wave therapy (ESWT) for frozen shoulder, demonstrating its efficacy and safety. Alessandro S *et al*. (19) analysed published literature and identified low-level laser therapy (LLLT) as the most effective modality among physical therapy interventions. Ping Lin *et al*. (20) reported that proprioceptive neuromuscular facilitation (PNF) represents an evidence-based approach for managing scapulohumeral periarthritis. Zhang *et al*. (21) investigated a modified arthroscopic outside-in shoulder release technique, showing significant improvements in joint range of motion and pain relief, establishing it as a suitable surgical option for severe shoulder stiffness. Minh DN *et al*. (22) demonstrated that bee venom acupuncture (BVA) surpasses intramuscular vitamin B1 injections in reducing pain, enhancing motor function, and normalising inflammatory cytokine profiles. Li *et al*. (23) confirmed that arthroscopic coracohumeral ligament release effectively alleviates early-phase shoulder pain, restores functional mobility, and addresses refractory cases of scapulohumeral periarthritis.

MUA has been demonstrated to be a safe and effective method for the treatment of frozen shoulder (24, 25). The primary principle involves inducing complete muscle relaxation and analgesia in the shoulder region, typically achieved through general anaesthesia or regional nerve blocks (e.g., brachial plexus block). Under these conditions, the physician performs a series of controlled, progressively forceful manual manoeuvres (including forward flexion, abduction, external rotation, and internal rotation) to mechanically disrupt thickened, contracted adhesions and fibrotic bands within the joint capsule, thereby restoring the normal range of motion of the glenohumeral joint. A prospective, single-centre randomised controlled trial demonstrated that, for stage II adhesive capsulitis, MUA can be considered safe compared to short-term physical therapy alone. Furthermore, MUA resulted in faster restoration of shoulder range of motion (ROM) and improved functional outcomes (26). Long-term follow-up (mean 13.2 years) of 398 patients with frozen shoulder (FS) who underwent MUA, conducted by Fairclough et al., revealed that 71.3% were asymptomatic [Oxford Shoulder Score (OSS) 48], 16.6% exhibited minor symptoms (OSS 42–47), and 12.1% reported significant symptoms (OSS <42). Self-reported recurrence rates exceeding 5 years post-initial MUA were 1.7% (4/240), with 0.8% (2/240) undergoing repeat MUA. At long-term follow-up, 6.7% developed rotator cuff pathology and 3.8% developed shoulder osteoarthritis (OA). This study demonstrates favourable long-term outcomes following MUA for FS (24). However, due to the inherent difficulty in precisely controlling the magnitude and extent of force application, MUA carries risks of iatrogenic complications such as capsular haematoma, brachial plexus injury, periarticular soft tissue damage, and fracture (27, 28). Furthermore, a significant risk of symptom recurrence exists. A prospective study found that synovitis was present in all 28 patients who received MUA before undergoing arthroscopic capsular release. Furthermore, an anterior capsular tear was identified in 27 of these patients. MUA induces capsular rupture as part of its therapeutic mechanism, which aligns with the intended clinical objective. Nevertheless, the anatomical location of the capsular tear is influenced by the specific technical manoeuvres employed during the procedure (29).

ACR represents the current gold-standard minimally invasive surgical intervention, particularly indicated for patients in the stiff/frozen phase of primary adhesive capsulitis. This procedure involves the use of specialised instruments (e.g., radiofrequency ablation devices, basket forceps) under direct arthroscopic visualisation to precisely and controllably release contracted, hypertrophic capsular and ligamentous structures, thereby alleviating mechanical obstruction and restoring normal shoulder ROM. Compared to traditional MUA, ACR offers the core advantage of “visualised intervention”, allowing targeted treatment of specific pathological regions while mitigating the risks associated with blind, forceful manipulation. Studies indicate that the majority of patients with frozen shoulder undergoing ACR achieve clinically meaningful outcomes within a 4-month timeframe. Most patients reach the minimal clinically important difference (MCID) threshold on outcome measures within the first postoperative month or two and attain a patient-acceptable symptom state (PASS) within 2–4 months postoperatively (30). A meta-analysis indicated that for the treatment of refractory frozen shoulder (FS), arthroscopic capsular release (ACR) demonstrated comparable short-term outcomes to MUA in terms of pain relief, functional improvement, range of motion (ROM) improvement, rates of mild complications, and the need for additional interventions. However, ACR was associated with a higher risk of severe complications (31). Another meta-analysis indicated that, compared to MUA, ACR demonstrated superior forward flexion outcomes at 3 and 6 months, while ACR and MUA showed no significant differences in VAS for pain, external rotation, or adverse events (32). Gábor Skaliczki et al., in a study of 59 patients with early primary frozen shoulder, concluded that arthroscopic capsular release is more effective than conservative management in alleviating pain (33). Nevertheless, ACR also presents certain limitations. It can be challenging to accurately assess the functional ROM endpoints intraoperatively (34). Additionally, technical difficulties arise from operating within a narrow joint space, potentially leading to incomplete release with residual functional limitations. Conversely, excessive release poses a risk of glenohumeral joint instability (35).

In this study, the treatment group received combined five-step MUA with arthroscopic release and intra-articular debridement, followed by cocktail anti-inflammatory analgesia. Compared with the control group, this approach offered dual advantages: 1) Staged release strategy: Initial manual mobilisation under anaesthesia gently lysed multi-directional adhesions, reducing the risk of violent manipulation-related complications (e.g., fractures, rotator cuff tears) inherent in pure MUA. 2) Arthroscopic supplementation: Targeted release of residual adhesions in limited motion directions was performed under direct visualisation, minimising operative time while allowing intraprocedural assessment of MUA-related injuries. This synergistic approach achieved comprehensive joint decompression, reduced complication rates, and resulted in lower 6-month recurrence rates and improved shoulder function scores. Notably, the combined procedure has inherent trade-offs: increased intraoperative bleeding due to pre-arthroscopic manipulation, higher technical demands for novice arthroscopists, and longer operative time with higher hospitalisation costs. These findings highlight the need for standardised protocols to optimise benefits while mitigating risks in clinical practice.

This study has several limitations that warrant consideration. Firstly, the retrospective design and limited sample size may reduce statistical power, underscoring the need for future large-scale studies to enhance analytical robustness and corroborate the current findings. Secondly, the non-randomised allocation of participants and variability in surgical operators across cases may have introduced selection bias and inter-operator variability, potentially confounding the estimation of treatment effects. Differences in baseline characteristics between groups and variations in surgeons’ experience or technique may have influenced outcomes independently of the intervention, thereby limiting both internal validity and generalisability to other settings. Thirdly, the inherent constraints of a retrospective approach preclude definitive causal inference, necessitating cautious interpretation of the conclusions; broader prospective investigations are required to substantiate these observations. Fourthly, owing to the limited sample size, we did not perform subgroup analyses for specific populations such as those with diabetes, which may have introduced some bias into the results. Fifthly, it is underpowered for low-frequency dichotomous outcomes such as complications and recurrence. Consequently, the absence of a statistically significant difference in these events should not be interpreted as evidence of equivalence. Furthermore, the relatively brief follow-up limited the assessment of long-term intervention efficacy, underscoring the need for extended observation periods in future studies to evaluate sustained outcomes. Additionally, no formal *a priori* power calculation was performed; sample size was based on the availability of eligible patients, leaving uncertainty regarding statistical power to detect smaller effects and interpreting the findings. In summary, although these preliminary findings provide valuable insights, their generalisability and long-term therapeutic impact should be confirmed in rigorously designed prospective trials with prolonged follow-up.

## 5 Conclusion

In summary, compared with five-step MUA alone, the combination of five-step MUA and ACR conferred modest but statistically significant advantages in pain reduction and ASES functional score at six months post-operatively. However, this approach was associated with increased operative time, postoperative hospital stay, and hospitalisation costs. Long-term outcomes remain to be established through further follow-up.

## Data Availability

The original contributions presented in the study are included in the article/Supplementary Material, further inquiries can be directed to the corresponding authors.

## References

[1] Codman EA. Tendinitis of the Short Rotators in the Shoulder. Rupture of the Supraspinatus Tendon and Other Lesions in or About the Subacromial Bursa. Boston: Thomas Todd (1934).

[2] Sarasua SM Floyd S Bridges WC Pill SG. The epidemiology and aetiology of adhesive capsulitis in the U.S. medicare population. BMC Musculoskelet Disord. (2021) 22(1):828. 10.1186/s12891-021-04704-9 34579697 PMC8474744

[3] Cho CH Bae KC Kim DH. Treatment strategy for frozen shoulder. Clin Orthop Surg. (2019) 11(3):249–57. 10.4055/cios.2019.11.3.249 31475043 PMC6695331

[4] King WV Hebron C. Frozen shoulder: living with uncertainty and being in “no-man’s land”. Physiother Theory Pract. (2023) 39(5):979–93. 10.1080/09593985.2022.2032512 35164645

[5] Lyne SA Goldblatt FM Shanahan EM. Living with a frozen shoulder—a phenomenological inquiry. BMC Musculoskelet Disord. (2022) 23(1):318. 10.1186/s12891-022-05251-35379207 PMC8978403

[6] Cho CH Song KS Kim BS Kim DH Lho YM. Biological aspect of pathophysiology for frozen shoulder. BioMed Res Int. (2018) 2018:7274517. 10.1155/2018/29992159 PMC5994312

[7] Lee HJ Yeomans DC. Opioid induced hyperalgesia in anesthetic settings. Korean J Anesthesiol. (2014) 67(5):299–304. 10.4097/kjae.2014.67.5.299 25473457 PMC4252340

[8] Sato H Droney J Ross J Olesen AE Staahl C Andresen T Gender, variation in opioid receptor genes and sensitivity to experimental pain. Mol Pain. (2013) 9:20. 10.1186/1744-8069-9-20 23570317 PMC3635934

[9] Yalcin N Uzun ST Reisli R Borazan H Otelcioglu S. A comparison of ketamine and paracetamol for preventing remifentanil induced hyperalgesia in patients undergoing total abdominal hysterectomy. Int J Med Sci. (2012) 9(5):327–33. 10.7150/ijms.4222 22745573 PMC3384914

[10] White PF Raeder J Kehlet H. Ketorolac: its role as part of a multimodal analgesic regimen. Anaesth Analg. (2012) 114(2):250–4. 10.1213/ANE.0b013e31823cd524 22266694

[11] Kraal T Lübbers J van den Bekerom MPJ Alessie J van Kooyk Y Eygendaal D The puzzling pathophysiology of frozen shoulders—a scoping review. J Exp Orthop. (2020) 7(1):91. 10.1186/s40634-020-00307-w 33205235 PMC7672132

[12] Challoumas D Biddle M McLean M Millar NL. Comparison of treatments for frozen shoulder: a systematic review and meta-analysis. JAMA Netw Open. (2020) 3(12):e2029581. 10.1001/jamanetworkopen.2020.29581 33326025 PMC7745103

[13] Rangan A Brealey SD Keding A Corbacho B Northgraves M Kottam L Management of adults with primary frozen shoulder in secondary care (UK FROST): a multicentre, pragmatic, three-arm, randomised controlled trial. Lancet. (2020) 396(10256):977–89. 10.1016/S0140-6736(20)31965-6 33010843

[14] Sundararajan SR Dsouza T Rajagopalakrishnan R Pushpa BT Arumugam P Rajasekaran S. Arthroscopic capsular release versus manipulation under anaesthesia for treating frozen shoulder: a prospective randomised study. Int Orthop. (2022) 46(11):2593–601. 10.1007/s00264-022-05567-y 36048234

[15] Lee SJ Jang JH Hyun YS. Can manipulation under anaesthesia alone provide clinical outcomes similar to arthroscopic circumferential capsular release in primary frozen shoulder? Clin Shoulder Elbow. (2020) 23(4):169–77. 10.5397/cise.2020.00311 PMC7726365 33330254

[16] Kraal T Beimers L The B Sierevelt I van den Bekerom M Eygendaal D. Manipulation under anaesthesia for frozen shoulders: outdated technique or well-established quick fix? Shoulder Elbow. (2021) 13(1_suppl):42–50. 10.1177/1758573220903171 PMC6440298 30993011

[17] Woods DA Loganathan K. Recurrence of frozen shoulder after manipulation under anaesthetic (MUA): the results of repeating the MUA. Bone Joint J. (2017) 99-B(6):812–7. 10.1302/0301-620X.99B6 28566402

[18] Cao DZ Wang CL Qing Z Liu LD. Effectiveness of extracorporeal shock-wave therapy for frozen shoulder: a protocol for a systematic review of randomized controlled trial. Medicine (Baltimore). (2019) 98(7):e14506. 10.1097/md.0000000000014506 30762780 PMC6408004

[19] De Sire A Agostini F Bernetti A Mangone M Ruggiero M Dinatale S Non-surgical and rehabilitative in patients with frozen shoulder: umbrella review of systematic reviews. J Pain Res. (2022) 15:2449–64. 10.2147/jpr.S371513 36016536 PMC9397530

[20] Lin P Yang M Huang D Lin H Wang J Zhong C Effect of proprioceptive neuromuscular facilitation technique on the treatment of frozen shoulder: a pilot randomized controlled trial. BMC Musculoskelet Disord. (2022) 23(1):367. 10.1186/s12891-022-05327-4 35443651 PMC9020070

[21] Zhang XC Liu K Ying H Yao G Fu XW Zhou BL A modified arthroscopic outside-in shoulder release approach for severe shoulder stiffness. Orthop Surg. (2023) 15(8):2167–73. 10.1111/os.13568 36321605 PMC10432466

[22] Nguyen M D Van Tran T Vinh Nguyen Q Doan Ha C Vu Phuong Dang L. Effectiveness of bee venom acupuncture for patients suffering from periarthritis humeroscapularis. J Tradit Chin Med. (2023) 43(4):795–800. 10.19852/j.cnki.jtcm.2023.04.002 37454265 PMC10320457

[23] Li DM Zhang C Xiang XX Cheng YF Zhang LF Ma K. The effect of arthroscopic extra-articular entire coracohumeral ligament release for patients with recalcitrant frozen shoulder. Orthop Surg. (2023) 15(8):1975–82. 10.1111/os.13566 36345115 PMC10432473

[24] Fairclough A Waters C Davies T Dacombe P Woods D. Long-term outcomes following manipulation under anaesthetic for patients with primary and secondary frozen shoulder. Shoulder Elbow. (2023) 15(2):173–80. 10.1177/17585732211070007 37035609 PMC10078811

[25] Pharr ZK Bowman EN Blickenstaff BE Hubler AK Brolin TJ Throckmorton TW Frozen shoulder manipulation with the FEAR technique: a retrospective case series with minimum two-year follow-up. J Surg Orthop Adv. (2022) 31(2):96–9. 35820094

[26] Kraal T de Wit Y The B van Boekel L Oost IK Boer R Improved range of motion after manipulation under anaesthesia versus physiotherapy for stage two frozen shoulder: a randomized controlled trial. JSES Int. (2023) 8(2):293–8. 10.1016/j.jseint.2023.11.004 38464443 PMC10920131

[27] Page MJ Green S Kramer S Johnston RV McBain B Chau M Manual therapy and exercise for adhesive capsulitis (frozen shoulder). Cochrane Database Syst Rev. (2014) 2014(8):Cd011275. 10.1002/14651858.Cd011275 25157702 PMC10882424

[28] Loew M Heichel TO Lehner B. Intraarticular lesions in primary frozen shoulder after manipulation under general anaesthesia. J Shoulder Elbow Surg. (2005) 14(1):16–21. 10.1016/j.jse.2004.04.004 15723009

[29] Mlv SK Mittal R Chauhan N. Arthroscopic findings after manipulation under anaesthesia in idiopathic capsulitis of the shoulder: a prospective study. World J Clin Cases. (2023) 11(34):8147–52. 10.12998/wjcc.v11.i34.8147 38130786 PMC10731179

[30] Pasqualini I Rossi LA Oyem PC Tanoira I Hurley ET Ranalletta M. Time required to achieve clinically significant outcomes after anteroinferior arthroscopic capsular release for shoulder adhesive capsulitis. Orthop J Sports Med. (2024) 12(11):23259671241275653. 10.1177/23259671241275653 39502374 PMC11536860

[31] Zhao Y Yang T Feng C Li L Pang L Zhao S. Arthroscopic capsular release versus manipulation under anaesthesia for refractory frozen shoulder: a systematic review with meta-analysis. Orthop Surg. (2024) 16(7):1517–29. 10.1111/os.14077 38747000 PMC11216839

[32] Xiao Y Tang H Meng J Wu Y Liu W Liu P Similar outcomes between arthroscopic capsular release and manipulation under anaesthesia for frozen shoulder: a meta-analysis. Asian J Surg. (2024) 47(10):4287–94. 10.1016/j.asjsur.2024.03.055 38531739

[33] Skaliczki G Kovács K Antal I Sallai I Kovács B Nyőgér Z Arthroscopic capsular release is more effective in pain relief than conservative treatment in patients with frozen shoulder. BMC Musculoskelet Disord. (2024) 25(1):145. 10.1186/s12891-024-07275-7 38365741 PMC10870563

[34] Sun JH Ruan WL Liu DK. Comparative effects of arthroscopic lysis and manipulation under anaesthesia for frozen shoulder. Clin J Med Off. (2017) 45:1240–3. 10.16680/j.1671-3826.2017.12.09

[35] Uppal HS Evans JP Smith C. Frozen shoulder: a systematic review of therapeutic options. World J Orthop. (2015) 6(2):263–8. 10.5312/wjo.v6.i2.263 25793166 PMC4363808

